# Highly Stable and Flexible Memristive Devices Based on Polyvinylpyrrolidone: WS_2_ Quantum Dots

**DOI:** 10.1038/s41598-020-62721-5

**Published:** 2020-04-01

**Authors:** Haoqun An, Yong Hun Lee, Jeong Heon Lee, Chaoxing Wu, Bon Min Koo, Tae Whan Kim

**Affiliations:** 1 Department of Electronic and Computer Engineering, Hanyang University, Seoul, 133-791 South Korea; 2 College of Physics and Information Engineering, Fuzhou University, Fuzhou, 350108 China

**Keywords:** Electrical and electronic engineering, Quantum dots

## Abstract

Tungsten disulfide (WS_2_) quantum dots (QDs) embedded in polyvinylpyrrolidone (PVP) based flexible memristive devices were prepared, and those devices exhibited typical bistable electrical switching and remarkable nonvolatile memristive behaviors. Maximum electricity ON/OFF ratio obtained from the current–voltage (I-V) curves of the device is close to 10^4^. The set voltage of the device is +0.7 V, which effectively reduced the energy consumption. The retention times extracted from data for the devices were as large as 1 × 10^4^ s, which points to these devices having nonvolatile characteristics. Moreover, the highly flexible characteristics of the devices were demonstrated by bending the devices. The carrier transport mechanisms were explained by fitting the I-V curves, and possible operating mechanisms of the devices can be described based on the electron trapping and detrapping processes. WS_2_ QDs uniformly dispersed in pure transparent N, N-Dimethylformamide (DMF) were obtained by using ultrasonication and a hydrothermal process in this work.

## Introduction

Over the past decade, hybrid inorganic/organic nanocomposites (HION) based thin film memory devices have been attractive subjects and have received great attention because of their superiority of low cost and power consumption, simple fabrication, which manifest their attractive and noteworthy applications in next generation memory cells, biomarker and artificial intelligence technologies, and logic computing^1–12^. Furthermore, HION-based memory devices are superior to other devices in the following aspects: the films are sturdy, and the active layers can be easily deposited. The devices can be manufactured using solution process and have both flexibility and mechanical strength, which are typical properties of polymer and inorganic materials, respectively^13–16^. Another advantage, which has attracted much attention, is that HION materials can be used as the active layers for bistable nonvolatile devices, with such devices exhibiting storage characteristics by trapping and releasing charges^1–4^. Among the various types of HION based memory device, nanoparticles and nanosheets of two-dimensional (2D) materials embedded HION memory devices are particularly attractive due to their properties of high flexibility and charge storage capability^2,4,5^. The appropriate energy levels and quantum confinement effects of 2D materials turn them into outstanding alternative materials for providing charge-trapping sites in memristive devices^9–12^.

As a typical 2D material, graphene has led to wide range of investigations on next generation electronic and optoelectronic systems due to its unique structure and optoelectronic properties. Many interesting applications of graphene, such as fuel cells, supercapacitors, hydrogen storages, and sensors, have been reported^17–19^. Because of the large number of reports on the achievements of graphene, research on tungsten disulfide (WS_2_) and molybdenum disulfide (MoS_2_), which are analogues of graphene, is once again receiving great attention. As a typical, nontoxic, semiconducting, transition metal dichalcogenide (TMD), WS_2_ has shown unprecedented promise in material science, with possible technological applications in optoelectronics, energy harvesting devices, chemical catalysts, sensors, and bio-markers^20–24^. The band gap of the WS_2_ can be changed from an indirect gap to a direct gap by exfoliating WS_2_ flakes to a lower-dimensional form of WS_2_, such as a nanosheet, which allows the WS_2_ material to exhibit remarkable properties^20,21^. The most noteworthy matter is that WS_2_ quantum dots (QDs) having more distinct and excellent optoelectronic properties due to its stronger quantum confinement effect than WS_2_ nanosheets^16–18^.

In this contribution, uniformly dispersed WS_2_ QDs with sizes of 10 nm were synthesized by using a combination of ultra-sonication and a hydrothermal exfoliating process. WS_2_ flakes were first exfoliated into nanosheets by ultrasonic treatment, and the WS_2_ QDs were synthesized by a facile hydrothermal process, which a means of cutting the precursor nanosheets placed in the autoclave in a high temperature and pressure environment^22–29^. N, N-Dimethylformamide (DMF), as an efficient catalyst for two-dimensional material exfoliating, has been briefly applied to the exfoliating of the WS_2_ flakes^28–34^. As a proof of our inference, we present a simple techniques for fabricating a memristive device that was embedded with synthesized WS_2_ QDs in a polymer matrix acting as an active layer and that exhibited excellent memory characteristics. The matrix material, polyvinylpyrrolidone (PVP), is a nontoxic, nonionic polymer having an excellent wetting property; it enable the fabricated device very flexible^5–16,35–38^. The nanomaterial of the active layer was synthesized by mixing the prepared WS_2_ quantum dots with PVP under laboratory conditions by a simply magnetic string process. In this investigation, the exfoliated WS_2_ QDs played important roles at charge-trapping sites, which resulted in the bistable electrical characteristics of the nonvolatile memory device. The nonvolatile rewritable properties of the device was investigated, and the possible conduction mechanisms were determined by several linear fitting models of measured I-V results. Also the device shows high stability and flexibility. We infer that the performance of this device is attributed to the extremely strong quantum confinement effect of WS_2_ QDs. These experiment results observed in this work provide valuable information for further investigations about 2D material QD-based organic bistable memory devices in the future^39–45^.

## Methods

Tungsten disulfide (WS_2_, 40–80 nm, 99.99%, amorphous) was purchased from 3302 Twig Leaf Ln, Houston, TX 77084, USA. Polyvinylpyrrolidone (PVP, NW-10000) was purchased from Aldrich Co. The suspension of WS_2_ dispersed N,N-Dimethylformamide (DMF) was prepared through a solution process, in a laboratory condition. We first added 10 mg of WS_2_ to 2.5 mL of DMF, the mixture was treated by sonicating for 5 h and then centrifuged for 20 min at 8000 rpm to remove the residual WS_2_ flakes at room temperature. Acquired suspension was transferred into a Teflon-lined stainless-steel autoclave and kept temperature at 200 °C for 10 h, subsequently cooled to room temperature naturally. The PVP powder was dissolved in liquid state DMF with a concentration of 10 mg/ml, and magnetically stirred the mixture for 3 hours in a room temperature afterward. A WS_2_ QD-PVP colloid with a WS_2_ QD/DMF colloid concentration of 0.8 wt%. The obtained solution was sequentially mixed by magnetic stirring process for 10 hours at an ambient of room temperature.

Memristive devices were fabricated by coating the synthesized WS_2_ QD-PVP nanocomposite on an indium tin oxide (ITO)-coated polyethylene glycol naphthalate (PEN) flexible substrates with a size of 2.5 cm × 2.5 cm. The WS_2_-PVP solution was deposited on the ITO film by spin coating at 300 and 3000 rpm for 20 and 40 s, respectively, and was annealed at 100 °C for 1 hour to evaporate the excess DMF. After annealing process, the Al electrodes with thicknesses of 200 nm were evaporated on the WS_2_ QD-PVP layer by utilizing a metal mask in a pressure of 1 × 10^−6^ Torr in a thermal condition.

The electrical performances were measured with the aid of Keithley 2400 Digital Source Meter. An external bias voltage was exerted to the Al electrode all the while, at the state of setting the ITO electrode ground. Atomic force microscopy (AFM) images were recorded by using a Dimension 3100 (Vecco, CA) system in the tapping mode. The photoluminescence (PL) measurements were recorded by using a spectrophotometer (Unico 4802). UV-vis spectra of synthesized WS_2_ QDs were investigated by utilizing a Shimadzu UV-3600 spectrometer. The image of Transmission electron microscopy (TEM) was carried out by a CM30 transmission electron microscope at a driving voltage of 300 kV. Ultraviolet photoelectron spectroscopy (UPS) imaging was carried out by using a Thetaprobe (UV source: He1, 21.2 eV; pass energy: 1 eV; energy step size: 0.05 eV).

## Results and Discussion

**Figure 1:**
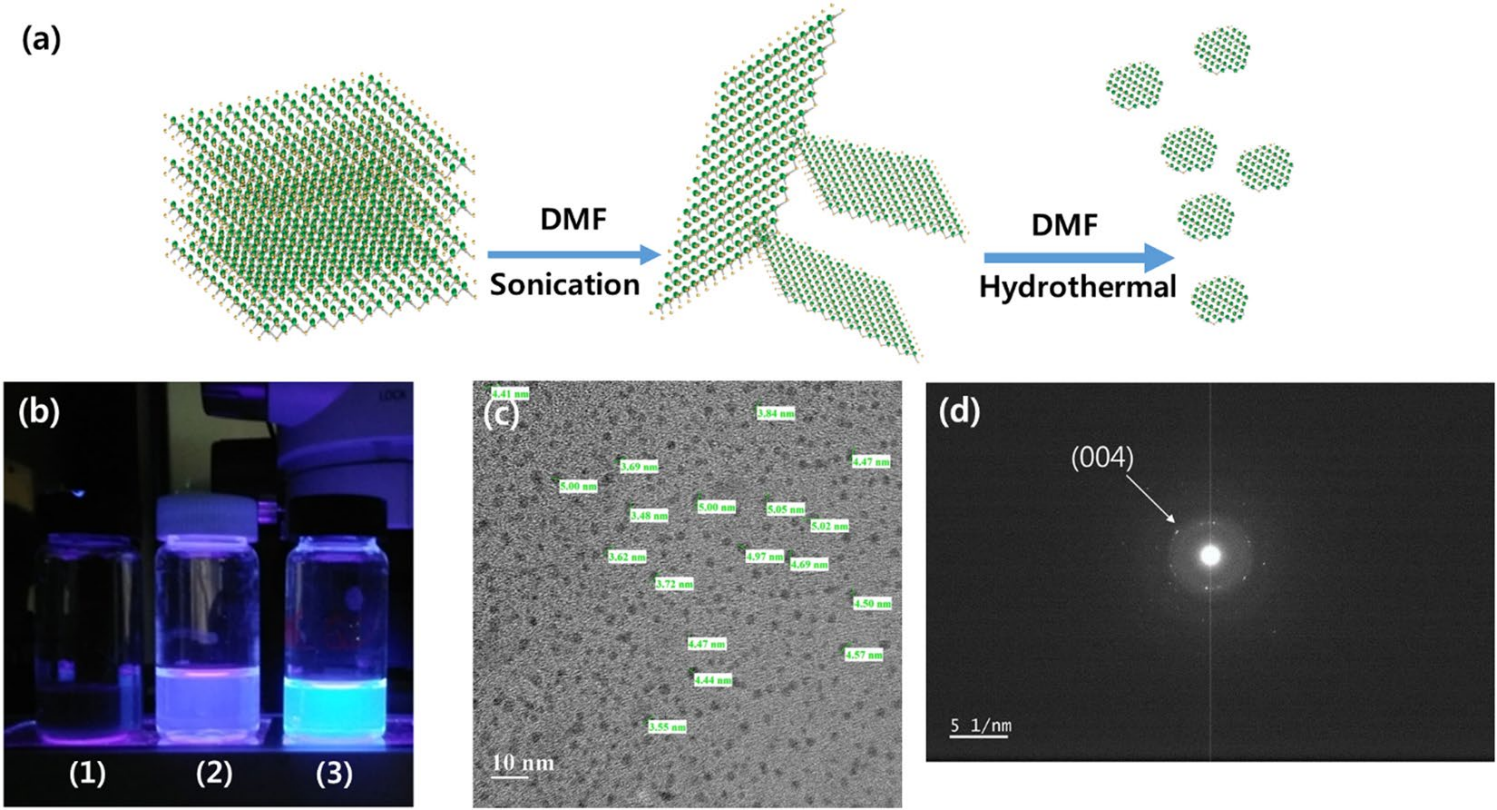
(**a**) Schematic illustration of catalyst-assisted exfoliation of WS_2_. After the WS_2_ flakes had been mixed with DMF, sonication was used to exfoliate the WS_2_ flakes to obtain a few WS_2_ nanosheets, and the hydrothermal method was used to obtain the WS_2_ QDs. (**b**) Photos of the solutions of exfoliated WS_2_ flakes in (1) ethanol, (2) DI water, and (3) DMF. (**c**) Transmission electron microscopy image of the WS_2_ QDs and (**d**) ED image of the WS_2_ QDs.

WS_2_ QDs with DMF-assisted exfoliation were prepared from WS_2_ flakes, and the WS_2_ QDs were successfully synthesized, as schematically shown in Fig. 1(a). In contrast, the WS_2_ flakes were also exfoliated in pure ethanol and de-ionized (DI) water, which can vaporize at high temperatures, leading to increased pressure in sealed autoclaves. The suspension obtained via the catalyst DMF exhibited high stability, but no precipitation could be observed even after it had been held at room temperature for several months. The experimental results shown in Fig. 1(b) clearly demonstrate that in a thermal environment, pure DMF can effectively exfoliate the layered WS_2_ flakes and disperse them into the catalyst. The TEM measurements on the diluted WS_2_ QDs solution, which were used to observe the appearance of the synthesized WS_2_ QDs on a nanoscale, were in progress. Figure 1(c,d) exhibits a distinctly dispersion of WS_2_ QDs and electron diffraction (ED) image, respectively. The observed specimens, as shown in the figure, were seen to express circular shapes and diameters ranging from 3 to 5.5 nm; the ED image manifestly shows the crystalline structure of the WS_2_ QDs catalytically synthesized by using pure DMF utilizing a combination of ultra-sonication and a hydrothermal process

**Figure 2:**
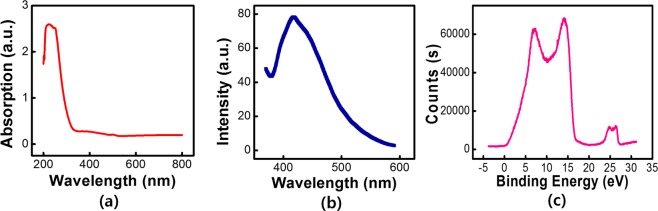
(**a**) Ultraviolet–visible–infrared absorption spectrum of the WS_2_ QD solution. (**b**) PL spectra of WS_2_ QDs dispersed in a DMF solution. (**c**) UPS spectra of WS_2_ QDs carried out by using a Thetaprobe (UV source: He1, 21.2 eV; pass energy: 1 eV; energy step size: 0.05 eV).

Figure 2(a) presents the ultraviolet (UV)-visible-infrared absorption spectrum of the WS_2_ QD solution taken under 365-nm UV light. Synthesized WS_2_ QDs dispersed suspension were clearly attested of high purity due to the unique single peak absorption value at the wavelength of 220 nm. The PL intensity emitted by the excited WS_2_ QD dispersion was investigated as a function of the excitation wavelength, and the results are shown in Fig. 2(b). Blue photoluminescent WS_2_ QDs were observed. An excitation-dependent PL is observed, and the peak is in reasonable agreement with the QD size distribution. The emission is seen to increase with increasing excitation until it reaches its maximum at an excitation wavelength of 420 nm, which according with the results for WS_2_ QDs manufactured via other approaches^23^. The excitation-dependent PL spectrum indicates polydispersity of the prepared WS_2_ QDs, which agrees with the characteristics of our synthesis method. The initial ultraviolet photoelectron spectroscopy (UPS) measurements were also obtained, the work function of the WS_2_ material is approximately 4.22 eV through the curve carried out, as shown in Fig. 2(c).

**Figure 3:**
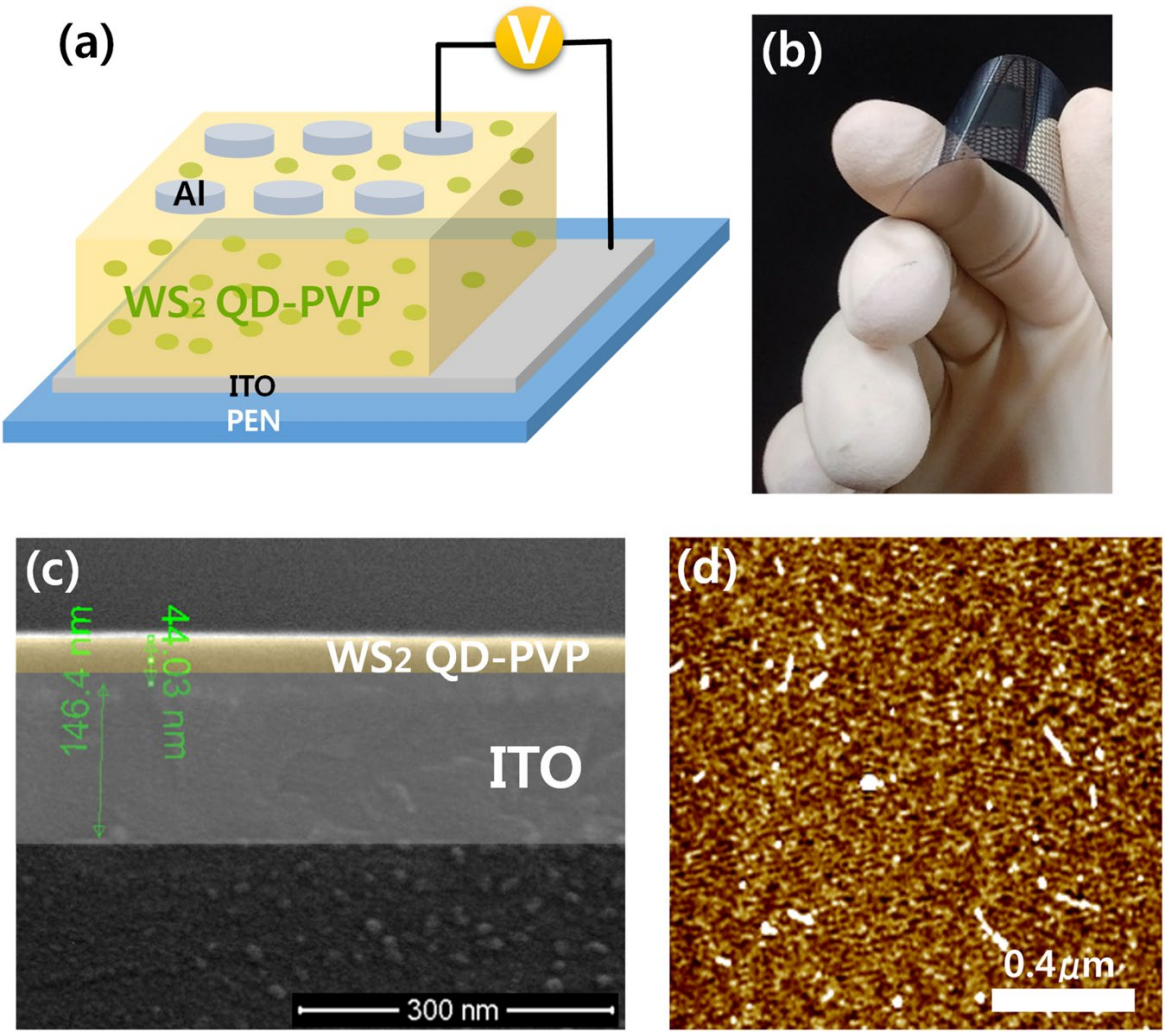
(**a**) Schematic diagram of the Al/WS_2_ QD-PVP/ITO PEN memristive device. (**b**) Photo of the Al/WS_2_ QD-PVP/ITO PEN memristive device. (**c**) Image of cross-sectional WS_2_ QD-PVP nanocomposite film uniformly coated on the indium-tin-oxide. (**d**) AFM image of the WS_2_ QD-PVP layer.

A schematic diagram of the device configuration with active layers and the practicality of the device for everyday use are shown in Fig. 3(a,b), respectively. Figure 3(c) shows a cross-sectional FE-SEM image of the WS_2_ QD-PVP nanocomposites formed on an ITO-coated substrate. The WS_2_ QD-PVP layer can be seen to have been uniformly coated on the ITO electrode. The image of atomic force microscopy (AFM) is shown in Fig. 3(d). An AFM determination was taken to characterize the surface roughness to characterize the deposited WS_2_ QD-PVP nanocomposites. The root-mean-square (rms) roughness of the WS_2_ QD-PVP nanocomposite layer is approximately 0.250 nm which strongly shows that the highly smooth and uniform WS_2_ QD-PVP active layer could be easily obtained by spin-coating process.

**Figure 4:**
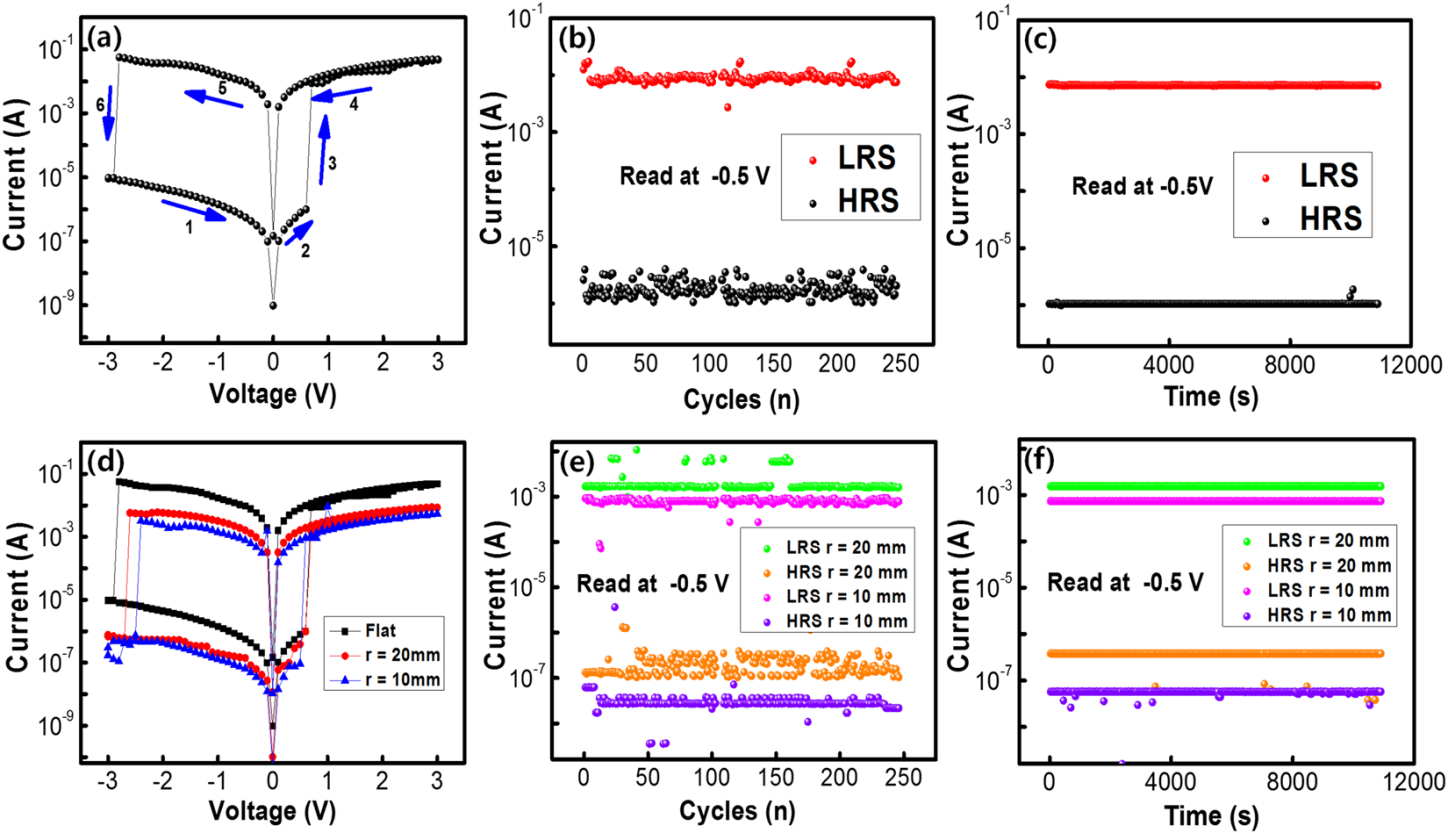
(**a**) Current-voltage characteristics of Al/WS_2_ QD-PVP/ITO/PEN memristive devices. (**b**) Current in the LRS and the HRS at −0.5 V for the endurance test. (**c**) Retention characteristics. (**d**) Current-voltage curves for the Al/WS_2_ QD-PVP/ITO/PEN devices for bending radius of 0, 10, and 20 mm. (**e**) Endurance measurements according to the bending cycles number of the device over 100 bending cycles under a reading bias voltage of −0.5 V. (**f**) Retention measurements after bending the Al/WS_2_ QD-PVP/ITO/PEN devices under a reading bias voltage of −0.5 V.

The memory effects of the Al/WS_2_ QD-PVP/ITO/PEN memristive device were characterized by using current-voltage (I-V) curves, and the results were exhibited in Fig. 4(a). Firstly, when the device was swept positively from 0 to +3 V, the current significantly increased with gradually increasing applied voltage, and increase abruptly from 1.0 × 10^−5^ to 1.0 × 10^−2^ A when the bias voltage increased to +0.7 V, indicating that the state of the device had transitioned from a high resistance state (HRS, which is denoted as an OFF state) to a low resistance state (LRS, which is denoted as an ON state). For the I-V curves, the process of device transition from HRS to LRS is seen as the ‘writing’ process in memory circuit cells, and an electricity ON/OFF ratio observed was as high as 10^4^. The features of our Al/WS_2_ QD-PVP/ITO/PEN memristive device promise low energy consumption and a low information loss rate during operation^35^. When the sweeping process in stage 1 and 2 applied again, the state of device can be maintained in LRS and even with the power was off, indicative of excellent stability and nonvolatile memristive characteristics of the memory device. Another attractive features of the device is that HRS of the device can be retrieved by applying a low negative voltage of −2.7 V, which is equivalent to the ‘erase’ process of a flash memory device. The process of second sweep shows electrical properties similar with the first sweep. Due to such properties of nonvolatile and rewritable, WS_2_ QD-PVP nanocomposites will allowed to be used as an electrically bistable material in flash memory devices.

Figure 4(b) exhibits the endurance performances of the devices. Cycling frequency in the process of measurements was approximately 0.3 Hz. No performance damping of the device occurred in the process of measurements. Results measured of retention characteristics on the LRS and the HRS for the devices were displayed in Fig. 4(c). Different resistance states remained a charge conservation for 10^4^ seconds under the normal condition, furthermore the observation that the conduction states were clearly distinguishable during that time indicative of the outstanding stability of the device.

In order to investigate the flexibility of the device, we bent the fabricated specimen so that its radii of curvature were 10 and 20 mm respectively, and the I-V results are shown in Fig. 4(d). I-V curve for the device under bending can be confirmed to be similar to that for the device without bending. No considerable variation in the ON/OFF current ratio was observed after the device had been bent. Also, as can be observed in Fig. 4(e,f), very few variation of the current values occurred by bended ITO electrode which slightly decrease the conductivity of the devices were seen during the endurance and retention tests respectively, indicative of the highly stable of the highly flexible memory device^13^.

**Figure 5:**
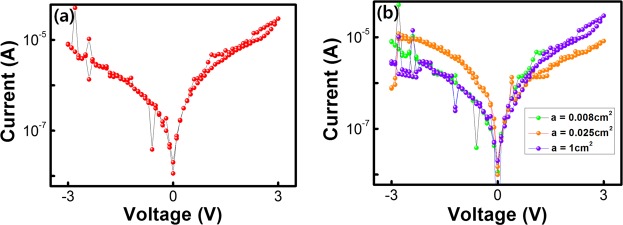
(**a**) Current-voltage characteristics of Al/PVP/ITO structure. (**b**) Current-voltage characteristics of Al/PVP/ITO structure with Al electrode areas of 0.008, 0.018 and 1 cm^2^.

Additionally, we point out that no bistability phenomenon was observed when a pure PVP layer without WS_2_ QDs was used. Figure 5(a) shows the I–V curves for pure PVP-based devices, and no bistability phenomenon is observed when an external sweeping bias is applied to the Al electrode. Furthermore, we investigated the influence of interface states at the junction of the PVP layer and the Al electrode, so we prepared pure PVP-based devices with different Al electrode areas, and we investigated their I–V characteristics^6,8,46^. Figure 5(b) presents the I-V curves for pure PVP-based devices with electrode areas of 0.008, 0.018 and 1 cm^2^; similarly, no bistability phenomenon were observed. Such results strongly demonstrate that the WS_2_ QDs play a key role in the electrical bistable behavior of our devices; accordingly, the charge trapping and releasing are attributed to the embedded WS_2_ QDs.

**Figure 6:**
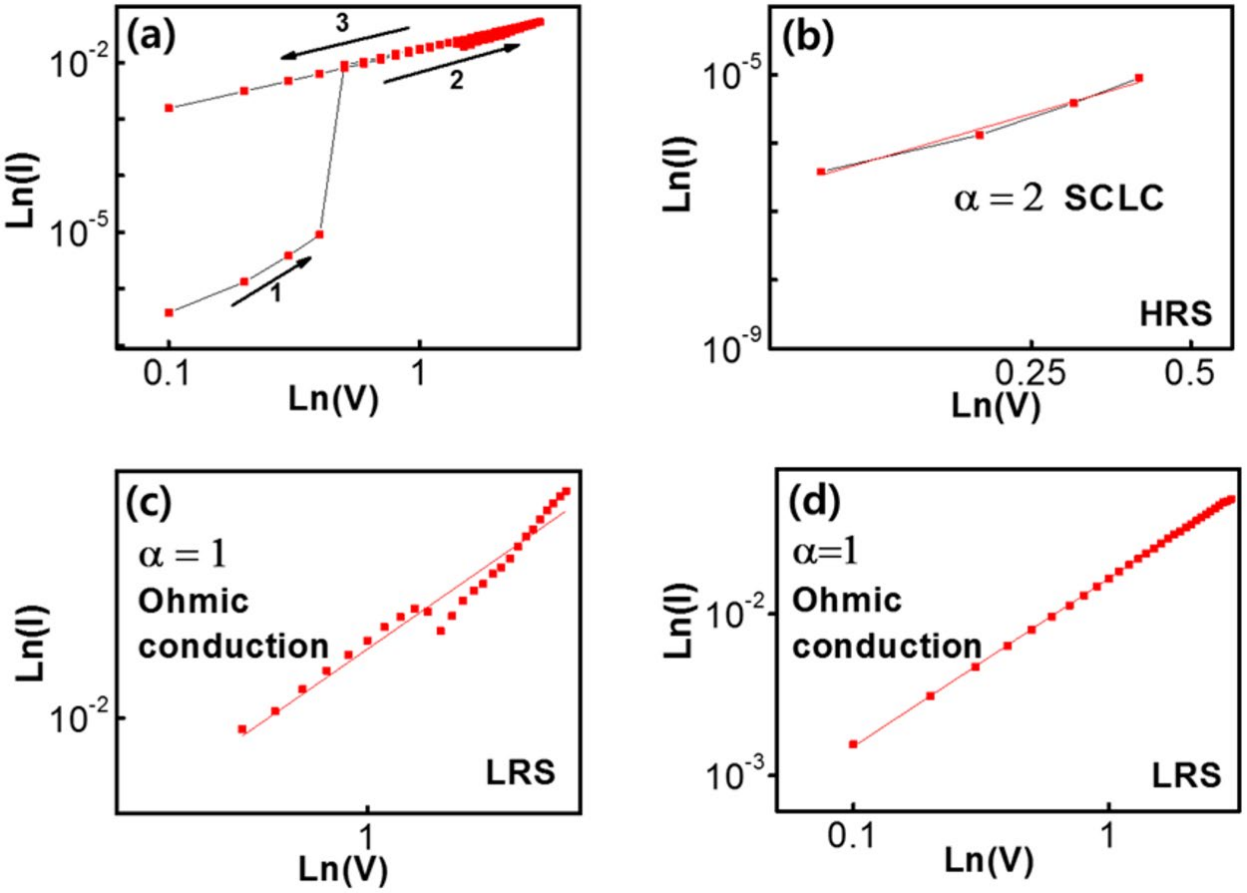
(**a**) Plots of the current obtained at a condition of applied voltage on the memristive device with a positive sweep. Fitting lines of the I–V curves in (**b**) region 1 in the OFF state, (**c**) region 2 in the ON state, and (**d**) region 3 in the ON state.

The obtained I–V data were fitted by using various conduction models to clarify the carrier transport mechanism, and the results are shown in Fig. 6(a). Fitting of the data was executed by utilizing two models, the space-charge-limited current (SCLC) and the Ohmic conduction models. Three distinct regions can be observed in the I-V plots, suggesting that a different conduction mechanism is operating in each region. In the OFF state, as the voltage applied was increased from 0 to +0.7 V, the current increased exponentially as I~V^m^ (m = 2.255) in the switching region 1 (shown in Fig. 6(b)). Such a linear characteristic on a ln-ln scale implies that the conduction mechanism in this process is most likely related to a space-charge-limited current, which can be fitted by using the following equation:

$$J=\frac{9\varepsilon \mu {V}^{2}}{8{L}^{3}},\,I\propto {V}^{2}$$

In the fitting equation, ε is the dielectric constant of the polymer (in this work, it’s PVP), *μ* is the mobility of the carriers in the active layer, *V* is the value of the external bias voltage, and *L* is the thickness of the active layer^47,48^. In the ln (I) ~ ln (V) of Fig. 6(c), a linear relation with a slope of 0.911 was observed in region 2 as the voltage was swept from +0.7 to +3.0 V, suggesting that Ohmic conduction was dominant. When the voltage was returned to the original applied voltage of 0 V, the plot of ln (I) ~ ln (V) remained nearly linear with a slope of 1.04, indicating the continuing dominance of Ohm’s law in region 3. The carrier transport at the LRS was not affected with the direction of the applied voltage due to the Ohmic conduction dominates for the LRS. It also indicates that the I-V curves for the LRS states in the voltages of positive range can be fitted by utllizing Ohmic conducting model completely, which displayed in Fig. 6(d).

**Figure 7:**
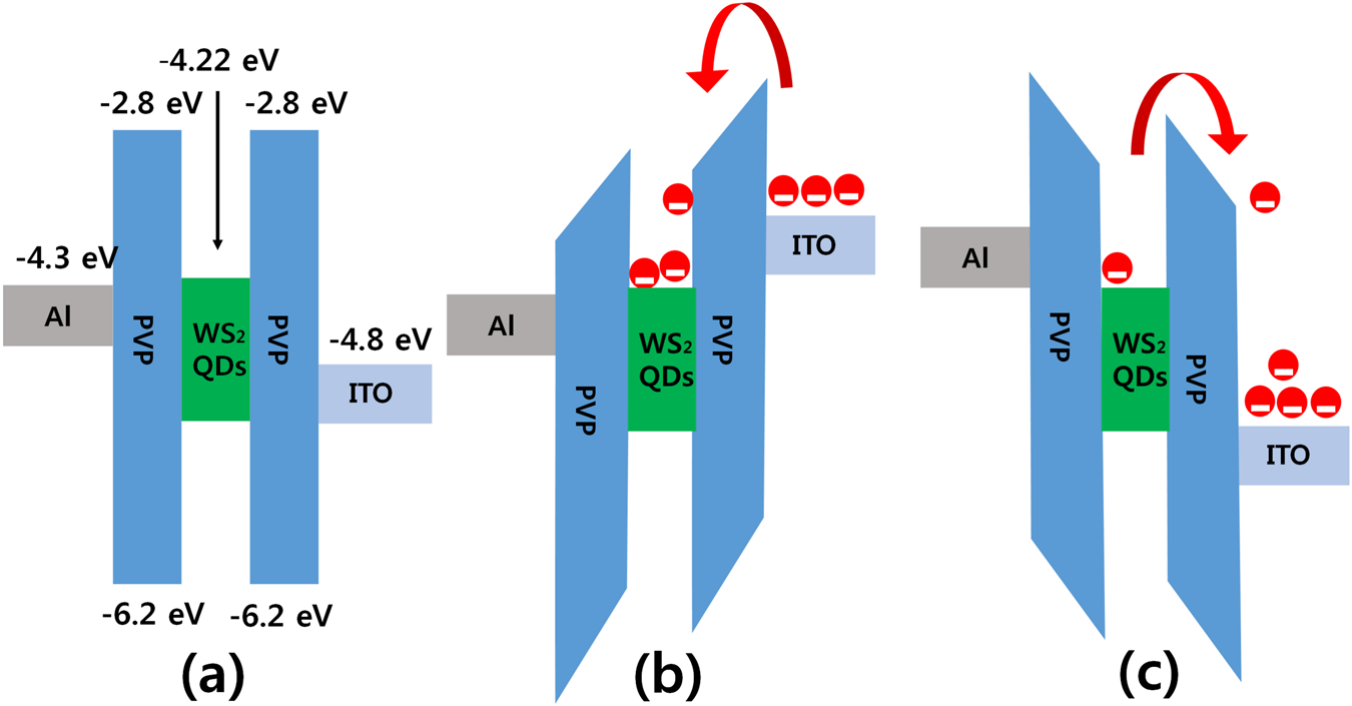
Schematic of the corresponding electrical band structures for the device operating under (**a**) zero bias and under (**b**) setting, and (**c**) resetting processes for the Al/WS_2_ QD-PVP/ITO memristive devices.

According to the results and discussions, we propose that the electronic transition between HRS and LRS can be explained by the mechanisms of carrier trapping and releasing behaviors of WS_2_ in the matrix of PVP. The proposed energy band diagram for the fabricated Al/WS_2_ QD-PVP/ITO device has shown in Fig. 7. The material work functions of the ITO and the Al electrode are −4.8 and −4.3 eV, respectively^11^. For the material of PVP, the highest occupied molecular orbital (HOMO) level is −6.2 eV, and the lowest unoccupied molecular orbital (LUMO) is −2.8 eV^23^. The electron affinity of the WS_2_ nanoparticles with a bandgap of 1.3 to 1.8 eV is low (−3.0 eV), which induces a large electron and hole injection barrier due to the applied voltage. The large barrier at the low voltage region (region 1 in Fig. 6(a)) results in a low injection of carriers. The carriers at higher voltage (region 2 in Fig. 6(a)) are injected into the PVP dielectric layer by tunneling through the barrier. The carriers are trapped by the WS_2_ QDs due to their having a lower energy level and to the quantum confinement effect. The amount of injected charge increases with any further increase in the applied switching voltage, leading to an abrupt current transition, at which time nearly all of the traps in the active layer are filled with charges. Subsequently, the device exhibits phenomenon of Ohmic conduction while the state get ON. Furthermore, the charges remained in the WS_2_ QDs were maintained even after cease the bias on the electrode, which accounts for the high conductivity and stability of our memory device. When a reset voltage is applied, the trapped charges are released. Then, the device returns to its initial HRS, which is called an erasing process; this indicates data storage has been achieved.

## Conclusions

A facile approach for exfoliating and dispersing WS_2_ flakes into a QD state with the aid of DMF has been reported. This approach provides a method for mass production of WS_2_ QDs stably dispersed in DMF. WS_2_ QDs stably dispersed in DMF and embedded in PVP exhibit high electrical bistability and flexibility, and we assume that the excellent performance of the devices can be attributed to the high quantum confinement of WS_2_ QDs; thus, this kind of nanocomposite can be used as an active layer in nonvolatile memristive devices. The thin-film memristive device based on the WS_2_ QD-PVP nanocomposite showed an electrically bistable behavior. The carrier transport mechanisms in the device may have resulted from the carrier trapping and releasing behaviors due to the trap sites formed by the WS_2_ QDs embedded in PVP organic matrix under the condition of applying external bias. This memristive device, by virtue of its nonvolatile and writable/erasable features, exhibits potential for applications in flexible organic memory cells, USB drives, bio-sensors, and other types of electronic and optoelectronic systems.
